# Study protocol: a cluster randomized controlled trial to assess the effectiveness of a multi-pronged behavioural intervention to improve use of personal protective equipment among migrant workers exposed to organic solvents in small and medium-sized enterprises

**DOI:** 10.1186/s12889-016-3268-6

**Published:** 2016-07-16

**Authors:** Wen Chen, Tongyang Li, Guanyang Zou, Xudong Li, Leiyu Shi, Shanshan Feng, Jingrong Shi, Fangjing Zhou, Siqi Han, Li Ling

**Affiliations:** Faculty of Medical Statistics and Epidemiology, School of Public Health, Sun Yat-sen University, 74 Zhongshan Road 2, Guangzhou, 510080 People’s Republic of China; Sun Yat-sen center for Migrant Health Policy, Sun Yat-sen University, 74 Zhongshan Road 2, Guangzhou, 510080 People’s Republic of China; Guangdong Prevention and Treatment Center for Occupational Diseases, 68 Haikang street, Xinhangxi Road, Guangzhou, 510300 People’s Republic of China; Department of Health Policy and Management, Bloomberg School of Public Health, Johns Hopkins University, 615 N. Wolfe Street, Baltimore, MD 21205 USA; Faculty of Health Management, School of Health Management, Guangzhou Medical University, 195 Dongfengxi Road, Guangzhou, 510182 People’s Republic of China

**Keywords:** Personal protective equipment, Migrant workers, Behavioural intervention, Cluster randomized controlled trial

## Abstract

**Background:**

In China, most of migrant workers work in the small and medium-sized enterprises (SMEs) and are a vulnerable group for occupational health. Migrant workers are at increased risk of occupational health risks due to poor occupational health behaviours such as the low use of personal protective equipment (PPE). However, there is a lack of solid evidence regarding how to improve the use of PPE among migrant workers in SMEs. The current study will assess the effectiveness of a multi-pronged behavioural intervention designed to promote PPE utilization among migrant workers exposed to organic solvents in SMEs.

**Methods/Design:**

This is a single blind, three-arm cluster randomized trial with 60 SMEs equally randomized to receive a top-down intervention (i.e. general health education and mHealth intervention provided by researchers) or a comprehensive intervention (which includes both top-down intervention and peer education) or a control condition (participants will not receive the intervention, but study measures will be obtained). Interventions will be conducted at the SMEs level for 6 months and all eligible migrant workers in these SMEs will be enrolled into the trial. The primary outcome is effective use of PPE during the last week. The secondary outcomes are occupational health knowledge and attitude and participation in occupational health check-up. Data will be collected and assessed at baseline; 3 months post baseline and the end of the intervention.

**Discussion:**

This theory- and evidence based intervention will contribute to the limited evidence of behaviour change intervention in improving PPE utilization of migrant workers in SMEs, and provide timely evidence for the development of basic occupational health services in China and elsewhere with similar industrialization contexts.

**Trial registration:**

ChiCTR-IOR-15006929. Registered on 16 August 2015.

## Background

A healthy workforce is essential for productivity and economic development [1, 2]. However, the burden of work-related diseases and injuries was high in developing countries. An estimated 2 million people die each year from work-related diseases causing an economic loss of about 2.8 trillion USD annually, or 4.0 % of the global gross domestic product (GDP) [3]. In these countries only 5 %–10 % workers have limited access to basic occupational health services aiming for the primary prevention and control of occupational and work-related diseases and injuries [4, 5].

In China, rapid industralisation has been associated with the rapid increase in internal population. Most of the migrants work in the small and medium-sized enterprises (SMEs), contributing to the tremendous economic development, but also exposed to great occupational risks and hazards. In China, the number of reported cases of occupational diseases have increased from 11,718 in 2000 to 29,972 in 2014, with an annual growth rate of 7.0 % [6, 7]. The majority of victims were migrant workers in SMEs [8]. It is estimated that about 100 million migrant workers in SMEs were exposed to occupational hazards [9]. However, due to their limited education, migrant workers tend to have poor occupational health knowledge, attitudes and behaviours. Lack of use of personal protective equipment (PPE) makes them prone to occupational diseases. Previous studies showed that among migrant workers in SMEs, only 16.8 %–22.4 % have used PPE effectively [10, 11]. There is an urgent need to improve the use of PPE, as a primary prevention to protect workers against occupational hazards, among migrant workers. Organic solvents rank as the top cause of chronic occupational poisoning, which is the second most common occupational disease in China [7, 8]. In this study, we aim to design a multi-level behavioural intervention to promote PPE utilization among migrant workers in SMEs who are exposed to organic solvents.

**Fig. 1:**
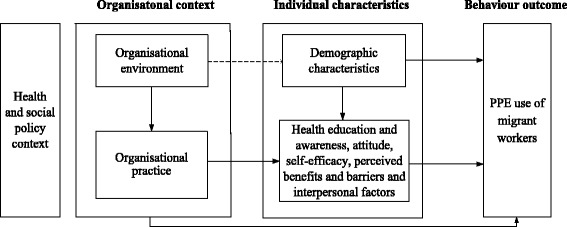
Conceptual model to reflect dynamics of factors in achieving the PPE use of migrant workers

Behaviour change cannot be achieved without first recognizing and understanding the complex factors that affect behaviours and the mechanisms by which migrant workers change and maintain their behaviours. Behavioural change theories, such as the Health Belief Model [12], Bandura’s Social Cognitive Theory [13], and the Theory of Reasoned Action [14], and Andersen’s Behavioral Model of Health Services Use [15], highlight the interaction of individual and contextual factors to influence the process of human behaviour change and health services use. In terms of workers’ PPE utilization, factors influencing the behaviour at the individual level include demographic characteristics [16], health education and awareness [17, 18], attitude towards the behaviour [19], self-efficacy, perceived benefits and barriers [16] and interpersonal factors [20]. At the organizational context level, factors can include implementation of regulation and occupational health service provision [21, 22] and an enabling environment to allow for effective PPE utilization behaviour [20]. Combining the factors at the individual and organizational levels, and based on behavioural models mentioned above and our exploratory study on the occupational health of migrant workers [10, 11], we proposed a conceptual model to reflect the dynamics of individual and organizational factors in achieving the behaviour outcome (Fig. 1). These variables are interrelated in a theoretically meaningful way, and have reciprocal effects on each other in predicting individual PPE use behaviour.

The intervention design will correspond to the conceptual model, addressing the barriers related to the influencing factors and PPE use behaviour. Traditionally, behavioural interventions have often been delivered in a passive and ‘top-down’ manner, such as health education by providing lectures, posters and leaflets. However, a previous review about effectiveness of occupational safety and health interventions has shown that general health education and training may not suffice to lead to occupational health behavioural change, especially for workers in SMEs [23]. The effectiveness of education programmes is compromised by the difficulties education providers face in reaching migrant workers in SMEs as a result of the limited access to workplaces, lack of trust, and cultural and/or literacy barriers. Over the last decade, mobile phones have widely been embraced as an integral part of our daily lives. Consequently, mobile Health (*mHealth*) interventions were applied to help to improve physical and mental health, especially for people who have barriers or limited access to health services [24, 25]. To our knowledge, mHealth intervention has not been developed in the field of occupational health for migrant workers in SMEs. Whether it works for occupational health behaviour change for migrant workers is one of questions we are going to answer in this trail.

In addition, a more bottom-up approach such as volunteers and peer support can empower individuals and maximize their potential to achieve behaviour change. Peer education was found to be effective at reducing HIV-related risk behaviours [26]. Peer supports are particularly important for migrants who tend to be less integrated with the local communities [27, 28]. Enhancing the support and education between workmates may help improve their social integration, develop positive social norms and promote healthy behaviour.

In this study, we aim to compare the effectiveness of a top-down intervention (which combines general health education and mHealth intervention provided by the research team) and a more comprehensive intervention (which includes both top-down intervention and peer education), on improving the use of PPE among migrant workers in the SMEs in China. Specifically, we will:

- Assess whether the intervention, either top-down or comprehensive, can significantly improve migrant workers’ knowledge, attitude, and behaviour of PPE utilization, compared to the control group.
- Examine whether the comprehensive intervention will be more effective than the top-down intervention.

## Methods/Design

### Design

**Fig. 2:**
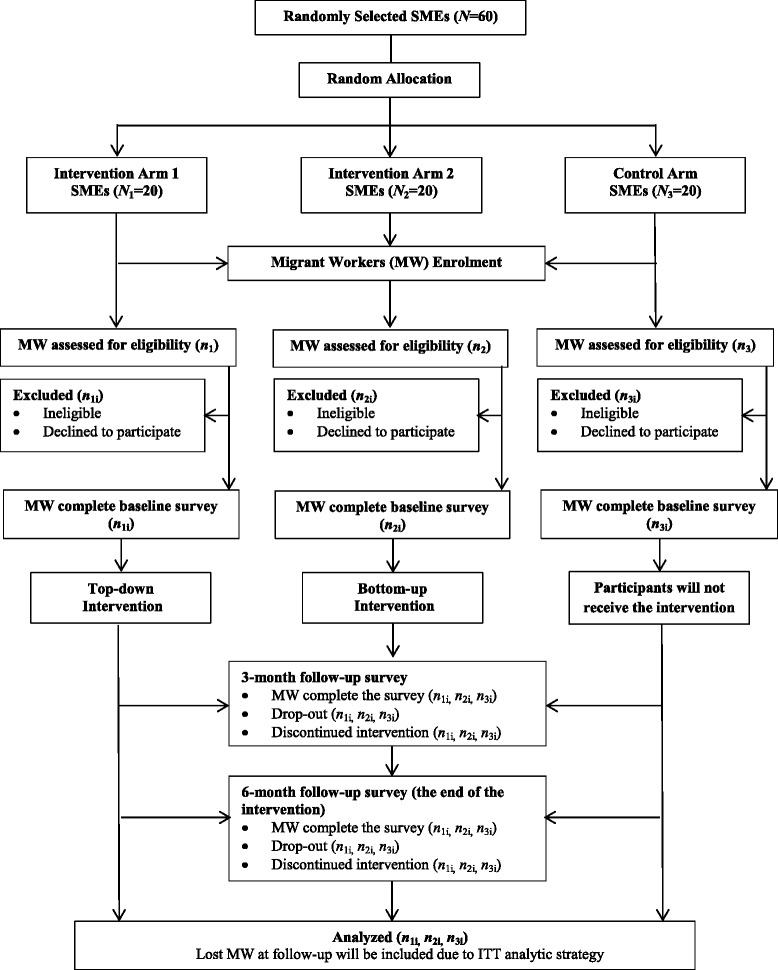
CONSORT flow diagram

This is a single blind, three-arm cluster randomized controlled trial (RCT) to be conducted in SMEs in China. A ‘cluster’ is defined as a SME, which is an enterprise employs between 300 and 1000 employees and has an annual turnover of RMB 20–400 million [29]. Outcome assessors will be blinded to the intervention or control status. Migrant workers will be the unit of analysis although we will take account of clustering by enterprises (Fig. 2).

### Study setting

This trial will be conducted in Baiyun district in Guangzhou. Guangzhou is one of the most industrially developed cities and a major destination for migrant workers in China. By the end of 2013, there were 8.37 million migrants in Guangzhou, accounting for 50.1 % of total population [30]. We will recruit 60 out of 861 SMEs where organic solvents are the major occupational hazard in Baiyun district as study settings. We select common organic solvents as the study parameter mainly for the following two reasons. First, organic solvents rank as one of the top occupational hazards in Guangzhou [31], as well as in China [7, 8]. Second, workers exposing to various kinds of organic solvents share similar working environments and use similar PPE, e.g. organic respirator and rubber gloves, across various enterprises, thus allowing for comparison across enterprises. In this study, organic solvents include but are not limited to benzene and benzene series, n-hexane, trichloroethylene, trichloromethane and dichloroethane.

### Recruitment

The recruitment process will be assisted by our collaborators, namely the local government and administration of work safety, who are responsible for occupational health and safety in the district. With a list of 861 SMEs where organic solvents are used in Baiyun District, provided by local administration of work safety, a total of 60 SMEs which meet inclusion criteria will be randomly selected by the researchers. Manager of these SMEs will be approached one week before the trail, with the help of our collaborators. An informed consent form that includes participant information sheet outlining the purpose, interventions, timing of assessments and main contents of questionnaires, will be sent to managers. SMEs agree to participate will be randomly allocated to one of three arms of the trial.

#### Inclusion criteria for enterprises

- Small and medium-sized enterprises;
- Organic solvents are constantly used in the production process;
- Providing workers with occupational personal protective equipment.

All available first-line migrant workers meeting inclusion and exclusion criteria will first be approached by their managers who will explain the purpose of the trial. We will then introduce main components of the trial again and ask them to sign a written consent form agreeing to participate in the trial.

#### Inclusion criteria for workers

- Migrant workers: workers without local registered permanent residence;
- The first-line production workers exposed to organic solvents;
- Working more than 1 month in the investigated enterprises;
- Written informed consent.

#### Exclusion criteria for workers

- First-line production team leaders;
- Not capable of accomplishing questionnaires due to reading or communicating obstacles.

### Randomisation

The unit of randomization will be SMEs. We chose an enterprise-level cluster randomized design to reduce contamination between the interventions and control conditions. Randomising migrant workers does not ensure migrant workers in the control arm will not receive any intervention information by working and communicating with others in the intervention arm. The Trial Statistician will use computer-generated random numbers to select 60 out of 861 SMEs where organic solvents are the major occupational hazard in Baiyun district and assigned them to intervention or control arms of the trial with a 1:1:1 ratio.

### Study interventions

The preliminary intervention plan was designed based on our previous exploratory study [20] and literature review on behavioural change theory and influencing factors of PPE utilization behaviour (Fig. 1). A qualitative study was then conducted in three SMEs to 1) provide details about the mechanisms through which individual and organizational factors affect the target behaviour; 2) understand the feasibility and acceptability of the proposed intervention; and 3) seek the suggestions of occupational health personnel and migrant workers on improving the intervention. Based on the interview results, the intervention plan was revised and finalized. We will test the effectiveness of intervention in three arms:

- ***Intervention arm 1*:** A top-down intervention including:
  - Occupational health education towards managers and occupational health personnel in each enterprise will be accomplished in the first week of intervention. The education will focus on enterprises’ responsibilities on occupational health; and benefits they will gain by creating a healthy workplace and improving workers’ health; as well as activities they could take to achieve the goal, e.g., providing appropriate PPE and establishing and enforcing a supervision plan. The education will be organized by local administration of work safety and delivered by trained educators.
  - General health education: One lecture on PPE utilization will be organised among migrant workers in the first week of intervention. The lecture will focus on the introduction of organic solvents, dangers of not using PPE, and how to properly select, use and store of PPE. The education in each SME will be delivered by two trained educators as a team. In addition, related brochures and posters will be delivered to migrant workers at baseline and 3-month follow-up of intervention.
  - mHealth intervention: PPE utilization and other related occupational health messages will be sent twice a week through Instant Message Apps, including WeChat, Tencent QQ and Fetion, depending on which App is more commonly used by each migrant worker.
- ***Intervention arm 2*:** A comprehensive intervention, including:
  - The same intervention as that in the intervention arm 1;
  - Peer education will be organised once a month. Each peer group will include 8–15 migrant workers and one of them will be assigned as a group leader based on the voluntary principle. Group leaders will receive a course on peer education and a handbook designed by the research team (WC, XL and SF), as well as establish contact with our project coordinators (TL, FZ, SH and JS) to send feedback and get help timely. The overall 6 monthly peer educations will be launched by group leaders for no longer than 60 min each time. The intervention will begin with an ice-breaking game and introduction of peer education in the 1st month, followed by organic solvents and the related protection education in the 2nd month, how to use PPE and personal experiences of benefits and barriers of use PPE in the 3rd-5th months, and maintenance of PPE utilization in the 6th month. In addition, group leaders will be asked to monitor other group members’ PPE utilization behaviour in the workplace. Every month, all group leaders will receive 50 RMB (8 $USD) and the top five best performing leaders will receive additional 50 RMB as a token of appreciation.
- ***Control arm*:** In the control arm, we do not provide any intervention, neither on the enterprises nor on the migrant workers. Occupational health circumstances at the workplace will be as usual.

This trial will last 6 months, and data will be collected at baseline, 3-month follow-up and 6-month follow-up (the end of the intervention), respectively (seeing Fig. 2 for flow diagram).

### Intervention fidelity

To maintain the quality of intervention delivery and track fidelity to the intervention, we will collect information from general health educators, peer education group leaders and migrant workers, respectively. First, general health educators will be asked to submit a written implementation form recording the adherence to protocol and photos via email to project coordinators as soon as the education was over. Second, at the end of each month, peer education group leaders will also be asked to submit an implementation form and photos to project coordinators to report the adherence to protocol, barriers to the implementation of peer education and their suggestions. PI, Co-PI and project coordinators of this project will look over these forms and provide feedback to all group leaders each month. Third, migrant workers’ self-reported adherence to the intervention, including the number of mHealth messages received each week, the number of education sections attended every month and their satisfaction, will be collected by the two follow-up questionnaire surveys.

### Outcome measures

The questionnaire data will be collected at baseline, 3th month follow-up and the end of intervention respectively. Baseline data will be collected prior to the research team starting the intervention. The three surveys will be carried out in both the intervention and control arms and organised with the help of enterprise mangers.

#### Primary outcome

The primary outcome is whether migrant workers have effectively used PPE during the last week, namely whether they have been wearing organic respirator, the most important PPE for organic exposure, when they are exposed to organic solvent. According to “The usage criterion of personal protective equipment against occupational diseases in organic solvents workplace” [32], workers should always wear organic respirator while exposed to organic solvents. Migrant workers will be asked to report the types of PPE, i.e. organic respirator, dust mask or surgical mask, they have used, and how often they have used them during the last week. Data administrator, who will not be involved in the implementation of the project, will determine whether migrant workers have effectively used PPE or not based on the types and frequency of PPE utilization. Given the lack of feasibility of long-term and continuous workplace observation and the potential for disrupting workflow and introducing additional biases, such as Hawthorne effect [33], the primary outcome will be measured through self-report method instead of observation by researchers. In addition, previous studies indicated that self-reported PPE use was highly consistent with field observation [34, 35].

#### Secondary outcome

1. Occupational health knowledge will be measured by 10 questions related to organic solvents and PPE utilization. Questions were designed by the researchers according to “The usage criterion of personal protective equipment against occupational diseases in organic solvents workplace” [32] (Cronbach alpha =0.81). Correct answer for each question will achieve a score of 1, giving a total possible score of 10.
2. Attitude towards PPE utilization. A scale of 9 items was developed by the researchers to assess migrant workers’ attitude towards PPE utilization. The scale includes four dimensions, i.e., willing of use, self-efficacy and perceived benefits and barriers (Cronbach alpha: 0.81–0.84 [20]). This measure will comprise 9 items and each will be rated on a 5-point Likert scale with ‘strongly agree’ scoring 5 and ‘strongly disagree’ scoring 1, giving an overall score ranging between 9 and 45.
3. Participation in occupational health check-up will be measured by whether migrant workers have taken part in occupational health check-up during the past 6 months, and the number of occupational health check-ups migrant workers have received.

### Data management

To avoid missing and logic mistakes, all completed questionnaires will be checked by the interviewers immediately. Questionnaires with missing items or obvious logical mistakes (e.g. not applicable items are filled in) will be returned to migrant workers to modify. Migrant workers and SMEs who are lost to follow-up will be recorded, and the reasons for workers’ and SMEs’ drop-out will be reported by enterprise managers and local administration of work safety, respectively. Before data can be analysed, data will be processed, including correcting data-inputting errors by using double data entry method and range and logic checks.

All personal information about enrolled participants will not be shared with any third party during and after the trial to protect confidentiality. Questionnaires will be stored in a locked file cabinet in a locked room. Data will be stored in a secure database with passwords encrypted.

### Sample size

We estimate the intervention will achieve an improvement of the primary outcome, i.e., ‘rate of effective utilization of PPE during the last week’ by 43 for top-down intervention arm and 53 % for comprehensive intervention arm. According to our previous study, among migrant workers exposed to organic solvents in SMEs, the rate of effective utilization of PPE was16.8 % [10]. Based on a pilot study we did in three SMEs in Guangzhou in 2014, we expected the rate will be increased to reach 60 and 70 % in top-down and comprehensive intervention arm, respectively. Based on an estimated intra-class correlation coefficient (ICC) of 0.05(previous literature reported that ICCs for participants’ behaviours and intent to change those behaviours were generally less than 0.1 [36]), an alpha of 0.05(one-side) and a beta of 0.20, we need 684 samples and 54 enterprises. We estimated the dropout rate will be 20 % as our exploratory study showed that annual turnover rate of migrant workers exposed to organic solvents in study SMEs was 26 %. In addition, considering that SMEs are sensitive to economic depression and based on experience of local administration of work safety, we also estimated 10 % of study SMEs may close down during intervention period. Therefore, we aim to recruit a total of 60 enterprises (20 for each arm) and at least 920 migrant workers.

### Data analysis

We will follow CONSORT guidelines [37, 38] to analyse and present the data. Intention-to-treat (ITT) analyses [39] will be used to estimate effectiveness of intervention. Therefore, all SMEs and migrant workers that are initially randomized and provide data at baseline will be included in the analysis. Migrant workers missing at baseline but present at follow-up surveys will be excluded from the ITT analyses.

The analysis will be conducted at the individual level. The baseline characteristics, e.g. demographic characteristics, occupational health knowledge and attitude and PPE utilization behaviour, of migrant workers will be described using mean (standard deviation), median (interquartile range) or proportions as appropriate and compared across three arms. Generalized estimating equations (GEE) will be used for the primary outcome adjusted for baseline PPE utilization behaviour to evaluate the effectiveness of intervention. A sensitivity analysis will be undertaken to adjust for the number of intervention sections received during the trial, which will be calculated based on general health educators, peer education group leaders and migrant workers’ feedback. In addition, subgroup analyses by migrant workers’ sex, age and education status, and SMEs’ occupational health practice, e.g. occupational health supervision, will be performed. For the secondary outcomes, linear mixed effects models will be applied for occupational health knowledge and attitude and GEE will be used for participation in occupational health check-up. Results from all secondary analyses will be treated as exploratory and presented as estimates with confidence intervals.

Although drop outs are expected, missing data will not be imputed. Because GEE and linear mixed effects models assume that missing data are missing at random. Under this assumption, the complete-case analysis by including predictors of missing observations will provide consistent estimates of the parameter [40].

## Discussion

Migrant workers are one of the main sources of labor force and a vulnerable group for occupational health. To our knowledge, this is the first behavioural intervention programme targeting PPE utilization of migrant workers exposed to organic solvents in SMEs in China. Our intervention is based on theoretical models, previous exploratory study and participants’ feedback. This study will contribute to the limited evidence on behavioural intervention for occupational health prevention in SMEs [23]. Our study will also contribute to the evidence base of developing basic occupational health services in China and elsewhere with similar industrialization contexts. In addition, this intervention has good replicability as they can be implemented by migrant workers themselves with relatively low cost. Ensuring the fidelity of a complex intervention is challenging, although we have developed stringent monitoring and reporting system. Sustaining the effects of behavioural change intervention will also be difficult. However, this problem will be mitigated given this is a theory-based pragmatic trial that also aims to strengthen the capacity of enterprise managers and workers.

## Abbreviations

GDP, Gross Domestic Product, GEE, Generalized Estimating Equations, ICC, intra-class correlation coefficient, ITT, Intention-to-treat, PPE, Personal Protective Equipment, RCT, Randomized Controlled Trial, SMEs, Small and Medium-sized Enterprises
